# A rare presentation of a bilateral intracranial parameningeal embryonal rhabdomyosarcoma mimicking vestibular schwannoma in a two-year-old child: a case report

**DOI:** 10.1007/s00381-022-05735-w

**Published:** 2022-11-10

**Authors:** Nadia Liber Salloum, Drahoslav Sokol, Jothy Kandasamy, Antonia Torgerson, Hamish B. Wallace, Chandrasekaran Kaliaperumal

**Affiliations:** 1 Department of Paediatric Neurosurgery, Royal Hospital for Sick Children, Edinburgh, UK; 2 Department of Neuropathology, Royal Hospital for Sick Children, Edinburgh, UK; 3 Department of Paediatric Oncology, Royal Hospital for Sick Children, Edinburgh, UK

**Keywords:** Rhabdomyosarcoma, Parameningeal, Malignancy, Intracranial

## Abstract

Intracranial parameningeal rhabdomyosarcomas are rare, aggressive, rapidly progressive paediatric malignancies that carry a poor prognosis. The authors report a case of a 2-year-old boy who initially presented with a left facial palsy, ataxia and, shortly after, bloody otorrhoea. MRI imaging was initially suggestive of a vestibular schwannoma. However, there was rapid progression of symptoms and further MRI imaging showed very rapid increase in tumour size with mass effect and development of a similar tumour on the contralateral side. A histological diagnosis of bilateral parameningeal embryonal rhabdomyosarcoma was made. Despite treatment, progression led to hydrocephalus and diffuse leptomeningeal disease, from which the patient did not survive. Few intracranial parameningeal rhabdomyosarcomas have previously been reported and these report similar presenting symptoms and rapid disease progression. However, this is the first reported case of a bilateral intracranial parameningeal embryonal rhabdomyosarcoma which, on initial presentation and imaging, appeared to mimic a vestibular schwannoma.

## Introduction

Rhabdomyosarcoma is the most common type of paediatric soft tissue tumour, accounting for approximately 6% of all childhood malignancies [1]. They typically occur in the first decade of life [1] and can occur anywhere in the body although approximately 40% originate in the head and neck [1]. Approximately half of those involving the head and neck can have parameningeal involvement [2, 3], typically arising from the nasopharynx, paranasal sinus, middle ear, infratemporal fossa and pterygopalatine area [3]. Histologically, rhabdomyosarcomas can be divided into four subtypes: embryonal, alveolar, botryoid and pleomorphic [4].

Presentation of parameningeal rhabdomyosarcomas can be non-specific and mimic other more common conditions, delaying diagnosis and therefore management [1, 5]. Symptoms can include sinusitis, otitis media, epistaxis, hoarseness, otorrhoea, aural polyp and cranial nerve palsies [1, 5]. Rhabdomyosarcomas of the head and neck are known to have a rapid onset and often present with advanced disease [6]. Parameningeal involvement can be associated with intracranial extension and skull base involvement which can make them inaccessible to complete surgical excision [1]. Intracranial parameningeal rhabdomyosarcoma tumours are rare but aggressive, fast growing tumours that carry a poor prognosis [1, 7, 8].

Here, we describe a rare presentation of a bilateral primary intracranial parameningeal embryonal rhabdomyosarcoma which initially mimicked a vestibular schwannoma.

## Case report

### History and examination

A 2-year-old boy with no significant past medical history or family history initially presented with a three week history of left infranuclear facial palsy and two day history of ataxia and vomiting. Initial imaging was suggestive of a vestibular schwannoma. Six weeks following this he represented with new onset blood stained otorrhoea and organic white debris in his left ear, at which point he was treated for otitis interna. Two weeks later, there was ongoing otorrhoea with new otalgia, cough, swallowing difficulties, drooling and hoarseness. The white debris noted in the left ear had increased in size to fully obstruct the ear canal. His ataxia at this time was persistent but not progressive and the facial palsy remained.

### Imaging

**Fig. 1:**
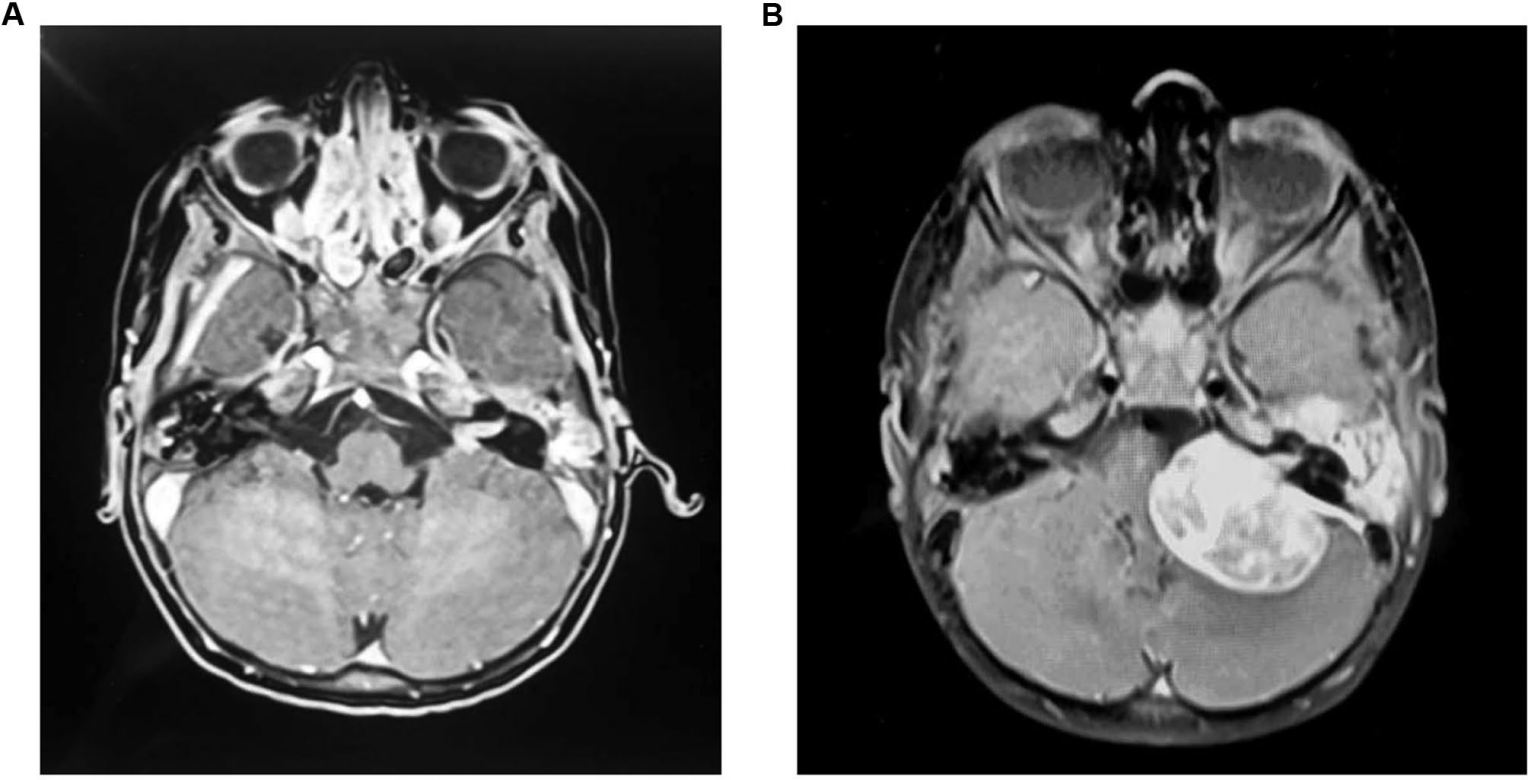
**a** Initial T1 post contrast MRI demonstrating 7 mm × 11 mm enhancing lesion in the left internal acoustic meatus **b** Follow-up T1 post contrast MRI demonstrating 4.0 cm × 4.0 cm × 3.3 cm on the left side and an enhancing lesion in the right temporal bone

Initial MRI imaging following the first presentation showed a lesion in the left internal acoustic meatus measuring 7 mm × 11 mm (Fig. 1a). There was no mass effect or hydrocephalus. Diffusion-weighted imaging (DWI) showed no diffusion restriction in the lesion. This was discussed at multidisciplinary meeting and initially thought to be an intracanalicular vestibular schwannoma for which follow up imaging was arranged.

Repeat MRI imaging was carried out at the time of representation with progression of symptoms, six weeks following initial imaging (Fig. 1b). This showed a rapid increase in the left sided mass, now measuring 4.0 cm × 4.0 cm × 3.3 cm, which was producing a mass effect on the adjacent cerebellum and brainstem. The left-sided lesion was noted to be centred on the left temporal bone at this time. There was also a new lesion noted in the right internal acoustic meatus measuring 3 mm × 5 mm. Diffusion-weighted imaging at this point also revealed no diffusion restriction in the lesions.

### Operation and histological diagnosis

**Fig. 2:**
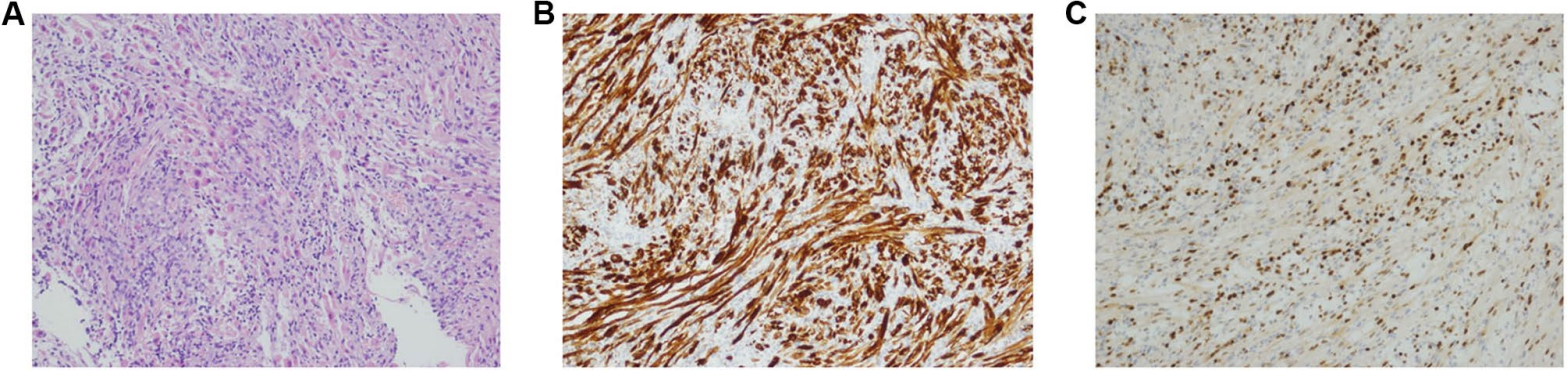
Histology slides: **a** Sections reveal elongated spindle cells and plump eosinophilic cells with a rhabdoid morphology (haematoxylin and eosin × 20). **b** Immunohistochemical staining with desmin shows strong expression (desmin × 20). **c** The rhabdoid cells show strong expression of myogenin (myogenin × 20)

A retromastoid approach on the left side was carried out with an aim to resect the lesion. Frozen section intraoperatively revealed elongated spindle cells and plump eosinophilic cells with a rhabdoid morphology contrary to the initial working diagnosis of vestibular schwannoma. Immunohistochemical staining was strongly positive for desmin and the rhabdoid cells were strongly positive for myogenin (Fig. 2). FOX01 FISH analysis shows no evidence of FOX01 gene rearrangement. A paediatric NGS solid tumour panel revealed no pathogenic variance causative of the reported phenotype. No gene fusions were detected using the RNA fusion panel phenotype. Fusion oncoprotein PAX3-NCOA2 analysis did not reveal any abnormality. The histology was in keeping with a high grade sarcoma with morphological and immunophenotypical features of a rhabdomyosarcoma. The final diagnosis was of a bilateral parameningeal embryonal rhabdomyosarcoma.

### Postoperative course

**Fig. 3:**
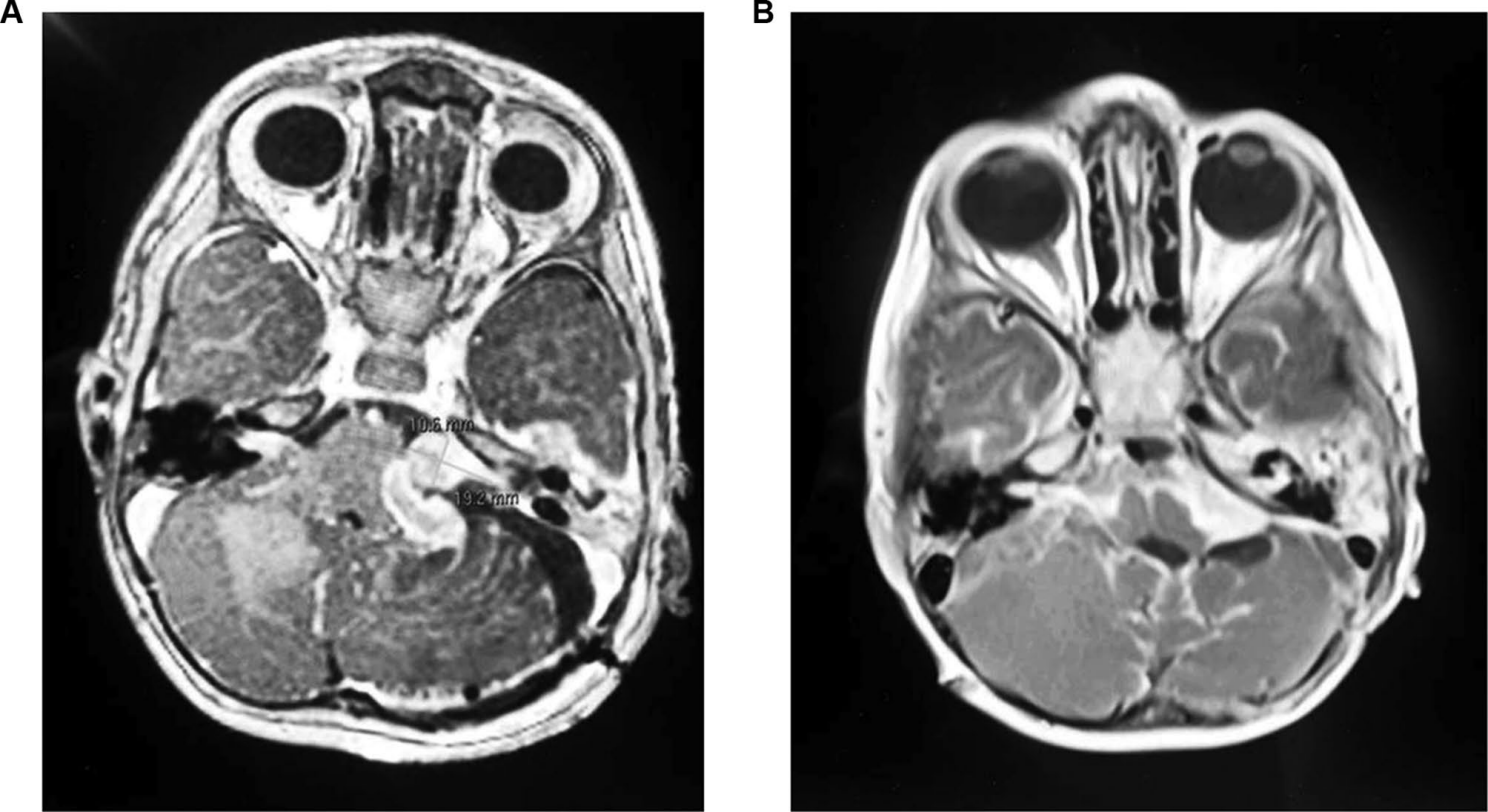
**a** Post operative MRI Brain with tumour remnant on the left side. **b** MRI demonstrating lesion progression after chemotherapy bilaterally

One week post operatively, the child developed new onset seizures. Further MRI imaging revealed new hydrocephalus due to cerebral aqueduct occlusion which was managed with surgical insertion of a ventriculoperitoneal shunt. MRI imaging at this time also revealed low grade osteomyelitis and cranial nerve palsies involving cranial nerves IV, VI and VII. There was progression of the right sided mass into the cerebellopontine angle and growth of the residual left-sided mass. There was enhancement of Meckel’s cave bilaterally (Fig. 3a).

Despite chemotherapy, the tumour progressed to diffuse leptomeningeal disease with compression of the lateral, third and fourth ventricles (Fig. 3b). Clinical symptoms progressed to include a right infranuclear facial palsy and ongoing seizures with mortality at 6 months following the initial presentation.

## Discussion

To date, we are not aware of any previously documented bilateral intracranial parameningeal embryonal rhabdomyosarcoma cases in the literature. However, several studies and case reports of rhabdomyosarcoma have been noted. Symptoms reported in these cases are reminiscent of those presented here, including facial nerve palsies, otorrhoea, ataxia and swallowing difficulties [3, 6, 9–11]. Of note, cranial nerve palsies have been found to occur in 50% [3] of parameningeal rhabdomyosarcoma cases.

Previous reports suggest symptoms and imaging were often initially indicative of another diagnosis such as vestibular schwannoma [9, 12] or otitis media [10, 13] prior to histological diagnosis. MRI or CT imaging alone is unable to differentiate intracranial rhabdomyosarcomas from some other tumour types, including vestibular schwannomas [14], and histological diagnosis is therefore required. This can contribute to diagnostic and management delays. Whilst reported symptoms in this case are characteristic for parameningeal rhabdomyosarcoma, the rarity of the condition in clinical practice and symptom overlap with more common conditions results in ongoing difficulty diagnosing this aggressive malignancy.

The case discussed here echoes the rapid progression described in the literature. Over a period of weeks, the tumour had rapidly increased in size and caused mass effect on several intracranial structures. It invaded the local bone and cranial nerves and led to hydrocephalus secondary to ventricular obstruction. Other reports have shown a similar progression from parameningeal rhabdomyosarcomas [9, 12, 15, 16].

## Conclusions

This is the first reported case of bilateral intracranial parameningeal embryonal rhabdomyosarcoma; an aggressive tumour with a poor prognosis. Presenting symptoms can easily mimic other more common, less rapidly progressive conditions such as vestibular schwannoma or otitis media. A high level of clinical suspicion is required to make a timely diagnosis and initiate treatment. Unfortunately, in this case, treatment was not successful in inducing remission and the bilateral tumours progressed rapidly to diffuse leptomeningeal disease.
